# Advertising Specialties

**Published:** 1868-02-15

**Authors:** 


### *Advertising Specialities.*

Communications are on file discussing this vexed question. Whether a card published in the secular press, announcing that M.D. so-and-so has such office hours at such a place, and pays especial attention to such and such a class, or species of affections, be, or not, guilty of violating the "code," and, therefore, entitled to condign punishment. The JOURNAL pauses before rushing into the melee.

